# Phase Studies of Additively Manufactured Near Beta Titanium Alloy-Ti55511

**DOI:** 10.3390/ma13071723

**Published:** 2020-04-07

**Authors:** Tuerdi Maimaitiyili, Krystian Mosur, Tomasz Kurzynowski, Nicola Casati, Helena Van Swygenhoven

**Affiliations:** 1 Materials and Process Development, Swerim AB, Isafjordsgatan 28A, 16440 Stockholm, Sweden; 2 Photons for Engineering and Manufacturing Group, Swiss Light Source, Paul Scherrer Institute-PSI, 5232 Villigen, Switzerland; 3 Centre for Advanced Manufacturing Technologies/Fraunhofer Project Center, Wrocław University of Science and Technology, ul. Łukasiewicza 5, 50-371 Wrocław, Poland; krystian.mosur@gmail.com (K.M.); tomasz.kurzynowski@pwr.edu.pl (T.K.); 4 Swiss Light Source, Paul Scherrer Institute-PSI, 5232 Villigen, Switzerland; nicola.casati@psi.ch; 5 Neutrons and X-rays for Mechanics of Materials, IMX, Ecole Polytechnique Federale de Lausanne, CH-1012 Lausanne, Switzerland

**Keywords:** titanium alloy, Ti55511, synchrotron, XRD, microscopy, SLM, EBM, EBSD, additive manufacturing, Rietveld analysis

## Abstract

The effect of electron-beam melting (EBM) and selective laser melting (SLM) processes on the chemical composition, phase composition, density, microstructure, and microhardness of as-built Ti55511 blocks were evaluated and compared. The work also aimed to understand how each process setting affects the powder characteristics after processing. Experiments have shown that both methods can process Ti55511 successfully and can build parts with almost full density (>99%) without any internal cracks or delamination. It was observed that the SLM build sample can retain the phase composition of the initial powder, while EBM displayed significant phase changes. After the EBM process, a considerable amount of α Ti-phase and lamella-like microstructures were found in the EBM build sample and corresponding powder left in the build chamber. Both processes showed a similar effect on the variation of powder morphology after the process. Despite the apparent difference in alloying composition, the EBM build Ti55511 sample showed similar microhardness as EBM build Ti-6Al-4V. Measured microhardness of the EBM build sample is approximately 10% higher than the SLM build, and it measured as 348 ± 30.20 HV.

## 1. Introduction

Additive manufacturing (AM) also known as “3D printing” is an advanced manufacturing technology which allow fabrication of geometrically complex and functional parts directly form the computer-aided design (CAD) model in a short time with limited tooling cost and with almost no material waste [1,2,3,4,5,6]. Hence, AM is a potential technology in areas where a high degree of customization and on-demand manufacturing is key such as aerospace and medical industry. 

Different types of AM techniques have been developed for metallic materials, and they can be categorized into many different subclasses based on energy sources, feed material, and ingredient material feeding approach as described by Liu et al. [7]. Among various AM techniques, the powder bed selective laser melting (SLM) and electron beam melting (EBM) are two common AM techniques, and these systems have been described in detail by Samy et al. [1] and Maimaitiyili et al. [2], respectively. In general, AM processes involve shaping a build plate and selectively melting the raw material (e.g., wire/powder) to form a three-dimensional solid object using a high energy focused laser or electron beam with multi-axis motion. Even though the basic operational principle of the AM method is relatively simple, the actual metal AM process is complex, and the results depend upon different settings of the system such as beam power (current), scanning speed, preheat temperature, etc. These are collectively referred to as processing parameters. In the AM process, processing parameters determine the build environment and cooling conditions and, consequently, affect the phase composition [1,2,8,9], residual stress [2], texture [2,8,10], surface roughness [11], density [7,12,13], and mechanical properties [3,7,12,13,14,15] of the as-built part. The lack of understanding of the relationship between process parameters and microstructure hinders the prediction of the properties of the built material and service life.

Titanium-based alloys have been widely used as an engineering material in many industries because of their excellent combination of a high strength/weight ratio and good corrosion resistance [1,2,10,12,14,15,16,17]. However, extracting high purity Ti and producing usable Ti-alloy parts are difficult and expensive processes. Therefore, there is a strong interest in using AM, such as SLM and EBM techniques, to process Ti-based materials. 

The mechanical properties of Ti-alloys depend on the microstructure, chemical, and phase composition [3,7,12,13,14,15]. Based on concentrations of alloying elements, Ti-alloys can be divided into three main classes: α, α + β, and (meta-stable and stable) β-alloys [17,18]. Depending on alloy composition and heating/cooling rates, Ti-alloys can also contain metastable α′ (hcp) and α” (orthorhombic) phases [1,17,18,19]. Therefore, it is of fundamental and technological importance to investigate the influence of AM processes on the phase composition and microstructure in Ti-alloys. Many AM studies have been performed on the two-phase (α + β) Ti-alloy known as Ti-6Al-4V [1,2,5,6,8,9,10,11,14,20] ,while other alloy compositions such as Ti–5Al–5Mo–5V–1Cr–1Fe (Ti55511) have been less addressed [13,21].

The Ti55511 is a near β-type alloy with important application in aerospace industries [13,22,23,24]. Compared to Ti-6Al-4V, the Ti55511 is a superior structural material, as it provides comparable or higher strength with 15%–20% less weight [24]. Most of our knowledge has been derived from conventionally manufactured materials [23,24]. Characterization of phases and microstructure after synthesis using different AM methods has not yet been performed. Here, we report and compare the build quality, microstructure, and phase composition of Ti55511 synthesized by SLM and EBM. 

## 2. Materials and Methods

### 2.1. Powders

Pre-alloyed Ti–5Al–5Mo–5V–1Cr–1Fe (Ti55511) powder prepared by gas atomization with an average particle size of 43 μm for SLM and 71 μm for EBM was obtained from KAMB Import–Export Warszawa (Nr CAS: 7440-32-6). The chemical compositions (wt%) of the as-received Ti55511 powder was Al 5.17, Mo 4.95, V 4.74, Cr 0.92, Fe 1.01, balanced by Ti. 

**Figure 1:**
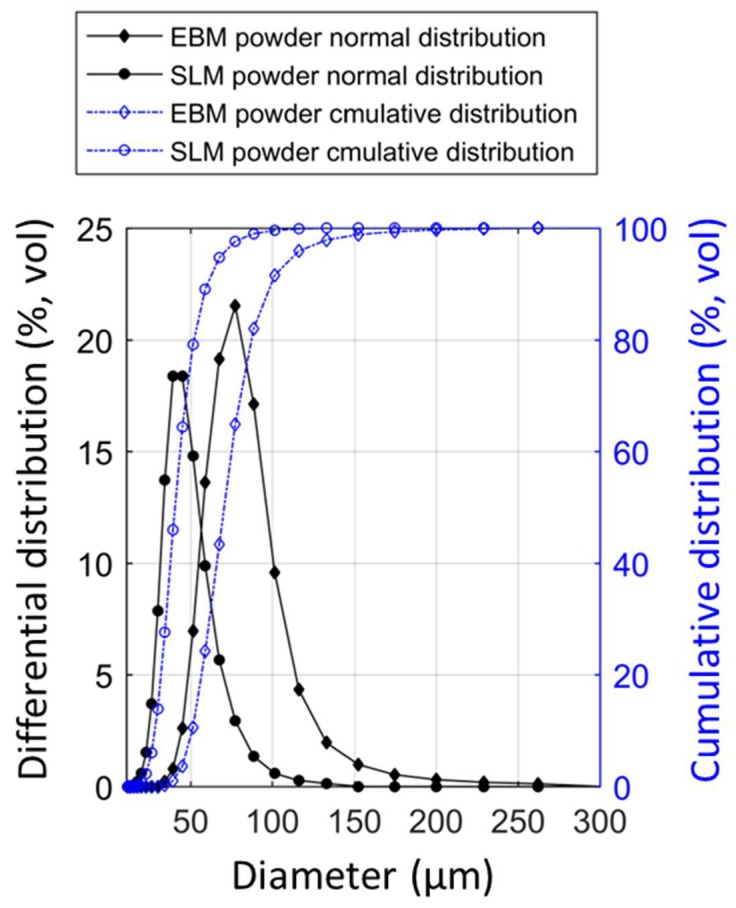
Particle size distribution of Ti55511 powders.

Laser diffraction particle size analyzer Partica LA-950 V2 system (Horiba, Tokyo, Japan) was used to measure the particle size distribution. For accuracy, each measurement was repeated three times. The results are plotted in Figure 1, and some important parameters are listed in Table 1. 

**Figure 2:**
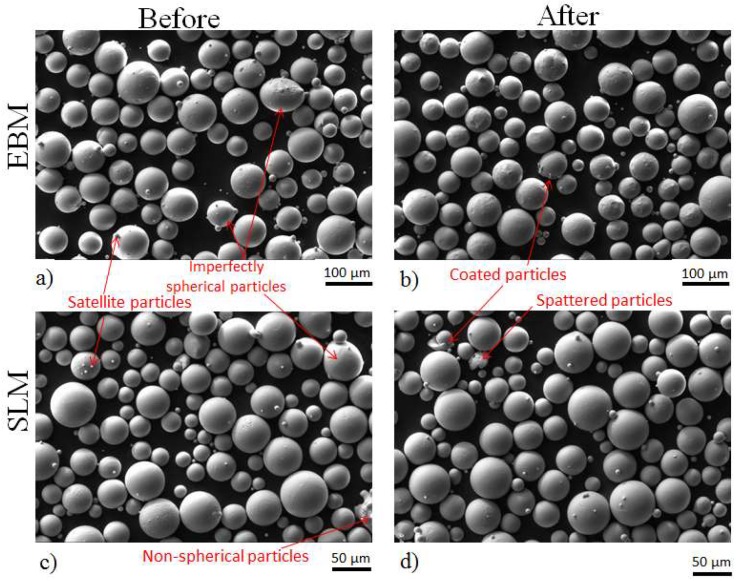
SEM images showing the morphologies of ingredient powder before (**a**,**c**) and after (**b**,**d**) the EBM (**a**,**b**) and SLM (**c**,**d**) processes.

**Figure 3:**
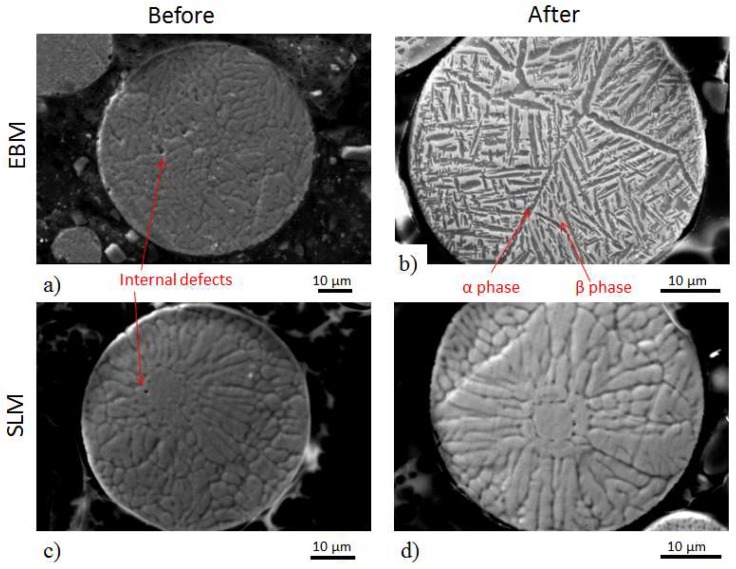
Internal microstructure of EBM powder (**a**) before and (**b**) after processing. Microstructure of SLM powder (**c**) before and (**d**) after processing.

A scanning electron microscope (SEM) equipped with an energy-dispersive X-ray detector system was used to examine the shape, size distribution, surface morphology, and external and internal defects of the powders. In addition, optical microscopy was employed to observe the internal porosity, defects, and cross-section of the powders. Selected SEM images of powders before and after processing are shown in Figure 2. Figure 3 shows the internal defect of the powders. The size distribution determined by SEM agrees well with data presented in Figure 1 and Table 1.

### 2.2. Sample Build Processes

The Ti55511 blocks with dimension of 2.5 × 2.5 × 5 cm were fabricated using both SLM and EBM methods in 90° (long side of the sample was along with the build direction), 0° (long side of the sample was in the build plane), and 45° (long side of the sample was 45° to the build plane) orientations. All microstructure related results presented were obtained from 90° samples. 

The SLM samples were made with ReaLizer 250 II SLM machine (ReaLizer GmbH, Borchen, Germany) equipped with a 400 W fiber laser. The laser melting process was carried under a protective argon atmosphere with O_2_ content less than 0.1 vol.%. The laser power (P) was 200 W, the scan speed (v) 330 mm/s, the hatch spacing (h) 0.21 mm, and layer thickness (d) 50 μm. To reduce thermal residual stress and elemental segregation, the samples were built on a Ti-6Al-4V substrate pre-heated to 250 °C prior to the building process.

The EBM specimens were built on an Arcam A2 machine (Arcam AB, Mölndal, Sweden) with a layer thickness of 50 μm. The processing parameters, such as spot size and scan velocity, were defined by the Arcam A2 process control algorithm. The scanning speed was 4530 mm/s, current 15mA, focus offset 3mA, and preheat temperature 650 °C. To investigate only the process effect, no post-treatment was applied to the specimens.

The scan strategy used in SLM was the “island” scan strategy in which each layer was divided into 3 mm × 3 mm square islands. Scan tracks in each island were exposed at the same orientation with respect to the neighboring island and rotated 90° among alternating layers.

In the EBM, a bidirectional scan strategy was used in which all tracks are made with alternating direction, i.e., left-to-right, then right-to-left.

The scanning strategies used are those that resulted in this material lowest residual stress and porosity which determined after cube print tests.

### 2.3. Characterization Methods

All samples used for microscopic studies were prepared by using standard metallographic preparation routines. Examination of microstructure was performed using a Visible Light Microscope (VLM, Leica DMRX + SpeedXT Core5, Wetzlar, Germany) and ZEISS NVision40 scanning electron microscope equipped with an energy-dispersive X-ray spectrometer (EDS) analysis system from Oxford Instruments (Oberkochen, Germany). To obtain phase and texture related information from the as-built material, electron backscatter diffraction (EBSD) investigations were performed using a field emission gun scanning electron microscope (FEG SEM) ZEISS ULTRA 55 equipped with an EDAX Hikari Camera (Oberkochen, Germany) operated at 20 kV in a high current mode with 120 μm aperture.

To identify the phase composition of powders, synchrotron X-ray powder diffraction were carried out at the Material Science (MS) beamline X04SA-MS4 of the Swiss Light Source (Paul Scherrer Institute, Villigen, Switzerland) using the MYTHEN II detector. All measurements were made at room temperature with 25.1 keV (λ = 0.4940 Å) X-ray beam and 60s exposure.

Diffraction data of as built material were acquired using a D500 X-ray diffractometer (XRD) from Bruker–Siemens (Karlsruhe, Germany) with Cu Kα radiation (λ = 0.15406 nm) operating at 40 mA and 40 kV. The step size and the acquisition time were 0.01° and 1 s respectively. All measurements were conducted at room temperature at the center of each test blocks cut from each sample faces (xy-, xz- and yz-planes) from the top- and bottom-half of the sample. The quantitative phase analysis was performed with a Topas-Academic software package.

Porosity was characterized using the Archimedes technique and microscopy. Cuboid samples were sectioned at different depths, ground and polished, and inspected in SEM. On average, 120 images were captured from each sectioned part and stitched with the functions in IMAGIC IMS V17Q4. These color images were then converted into 8 bit black-and-white images using ImageJ. To understand the shape of the pores, a circularity of the pores was also calculated with this program.

## 3. Results and Discussions

### 3.1. Powder Characterization

Figure 1 and Table 1 present the results of the powder size distribution (PSD) of different powder samples obtained using a Partica LA-950 V2 laser particle size analyzer (Kyoto, Japan). Figure 2 and Figure 3 show the morphology and external/internal defect of the powders, respectively. Both SLM and EBM powder particles predominantly in spherical shape with limited quantity of non-spherical particles and spherical imperfections. Both SLM and EBM powders have a nearly normal size distribution (Figure 1). The SLM powder had a size distribution between 28 (D10) and 60 µm (D90) with mean volume diameter around 40 µm. The EBM powder had a size distribution between 51 (D10) and 99 µm (D90) with mean volume diameter around 70 µm. 

It has been reported that a spherical powder with narrow PSD has a positive effect in both mechanical properties and finishing surface for the sample [25]. Because of charging problems associated with electron beam, commonly, a larger powder is used in the EBM [2]. According to the literature [2,4,5], measured PSD of the EBM and SLM was in the suggested PSD range for respective methods. Hence, both types of powders are ideal for processing with corresponding methods and all observation reported here can be directly related to the alloy and the manufacturing methods in use.

After processing, the powder remains predominantly spherical as shown in Figure 2b,d. Table 1 shows, however, that both the SLM and EBM process caused changes in powder size distribution. The averaged sizes tended to increase which can be due to the powder agglomeration or powder recoating. Occasionally, broken powder particles were observed as shown in Figure 2d. It is, however, clear that both process routes have limited effect on the powder quality.

Figure 2 shows cross-sections of embedded powder after etching before and after usage. The initial microstructure (before usage) of the SLM and EBM powders was very similar. The powder used in the SLM process was not different, but the powder used in EBM showed a lamellae microstructure with a mixture of very fine and coarser α and β phases. Similar lamella microstructure is reported by Li et al. [26] for thermally treated conventional Ti55511 which first solution treated at 920 °C for 120 min and annealed at 700 °C for 60 min. Therefore, it is believed that the high build plate temperature used in the EBM process together with heat dissipated from the melt zone during electron beam scanning is the main cause of such significant phase transformation observed in the remaining powders left in the build chamber.

The chemical composition of powder samples before and after the process was assessed by EDS analysis. As in Figure 3, the elemental map of powders before SLM and EBM process and as well as after SLM process do not show any distinct regions, and they all seemed homogeneous and featureless. However, there are two distinct regions in the elemental map of powder after the EBM process: Mo dense and Mo depleted region. Such a difference indicates a difference in phases. In Ti-alloy, Mo and V work as a β stabilizers, and Al is an α stabilizer [17]. Therefore, during phase formation, these elements will preferentially partition to the respective phases.

**Figure 4:**
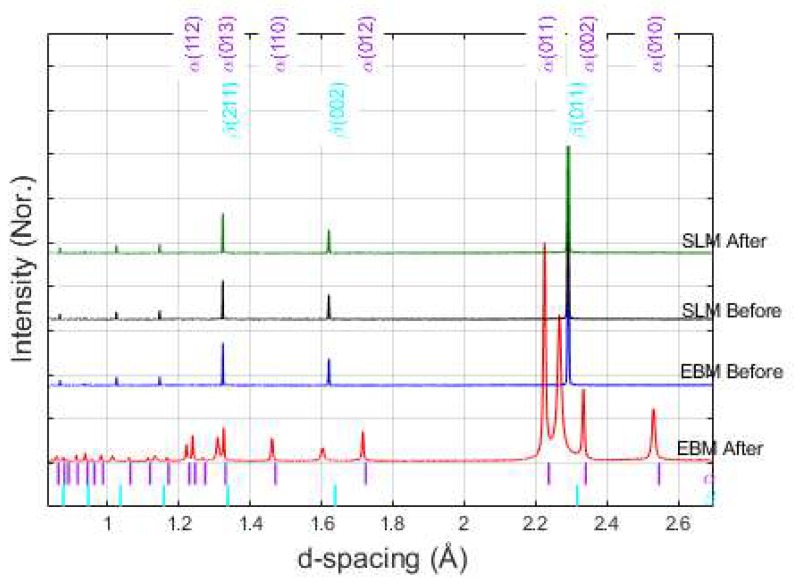
Diffraction pattern of powders before and after the process. These color-coded tick marks under the diffractogram correspond to the expected peak positions of α- and β-Ti phases reported in the Inorganic Crystal Structure Database (ICSD) [27] (ICSD reference number of α-phase is 191187 and β-phase is 653278).

The diffraction patterns of powders before and after the process shown in Figure 4 confirm a microstructure only composed of the β Ti-phase. Remaining powder in the SLM build chamber after the process consisted of comparable phase composition as ingredient powder, however, the remaining powder after the EBM process showed the clear presence of α Ti-phase in addition to β Ti-phase.

It is well known that titanium and its alloys have a strong affinity for oxygen, and they can react to form detrimental oxides at elevated temperatures which potentially degrade the quality of the build parts [7]. However, from Figure 4, one can see that all powders are free from oxides. In addition, all powders did not show any observable color changes after the process. Therefore, it is safe to say that both EBM and SLM process are equally effective in preventing oxygen contamination, and powder degradation related to oxygen from both methods are minimal.

It is commonly reported that the powder morphology [25], oxygen content [7], and PSD [25] have significant impact on the final build material quality, and for that reason these parameters are often used/discussed in the literature for evaluating the impact of an additive manufacturing process to the powder degradation behavior. However, it is not clear whether the phase composition of the ingredient powder has any influence on the porosity of the build parts. As different phases have a different crystal structure and each phase mixtures can have specific microstructures, it can be expected that the thermal/chemical properties of the powders with phase transformation can be different from the standard/initial powders. Therefore, it might be also important to include powder phase composition in the discussion of powder degradation evaluations together with other parameters.

### 3.2. Surface Roughness

**Figure 5:**
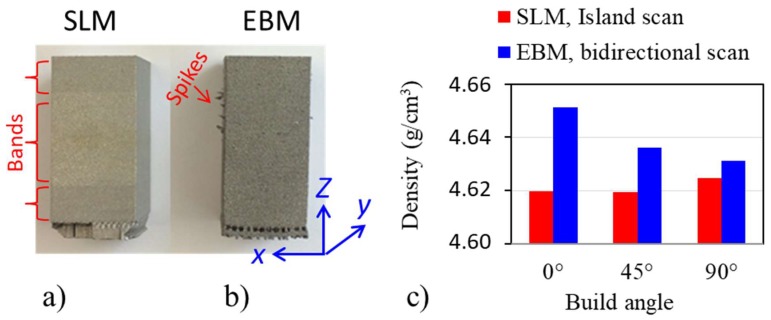
Photography of (**a**) SLM and (**b**) EBM samples build on 90° orientation. (**c**) Density comparison of SLM (red) and EBM (blue) samples built on three different orientations obtained from the Archimedes principle.

All builds from both methods were successful, with no warping, distortion, lifting from the base and no macro/micro-scale cracking. The physical appearance of vertically built samples in the 90° orientation from SLM and EBM are shown in Figure 5a and Figure 5b, respectively. Clear band-like patterns were observed in the SLM-built specimen (Figure 5a). A limited number of rough spikes sticking to the sides of the EBM-built specimen, as shown with arrows in Figure 5b, were observed. It is believed that these spikes might be caused by fallen agglomerated powders/melts which spatter out during scanning processes. As the distribution of these spikes is random, the occurrence is limited and can be removed relatively easily, they are excluded from the surface roughness evaluations. The roughness was measured with a Veeco Dektak 8 (NY, USA) profilometer and values of 12.27 μm and 38.05 μm were obtained for SLM and EBM, respectively. The surface roughness of the additively manufactured parts was mainly influenced by the powder particle size and build layer thickness [28,29]. In general, the smaller the particle size, the thinner the layer thickness and so the higher the surface quality. The size of the powder particles used in EBM was twice the size of SLM, so a higher surface roughness was expected for EBM.

### 3.3. Porosity

**Figure 6:**
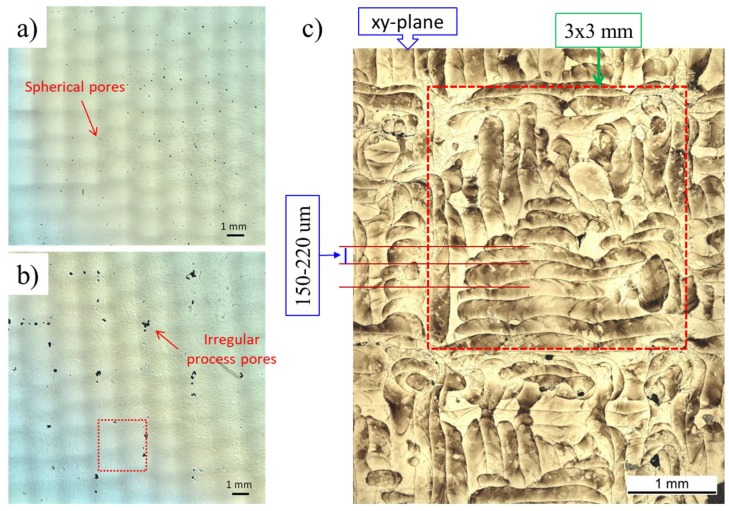
VLM image of (**a**) EBM and (**b**) SLM samples before etching. (**c**) VLM image of SLM sample after etching. All images are taken from the xy-plane (or build plane) of samples.

Figure 5c shows the Archimedes density measurement results of samples built in three different orientations. The standard deviations of density measurement were less than 0.005 g/cm^3^ for all measurements. As can be observed, EBM in general gave higher density than SLM irrespective of sample build orientation, but the difference between these two was less than one percent (0.39%). With respect to reported densities in literature, both methods can produce an almost fully dense structure (99.38 and 99.77% for SLM and EBM, respectively). Results of the porosity after image analysis from the xy- and xz-planes agreed well with the results presented in Figure 5c. The SLM sample had more pores than the EBM samples. The size of the pored ranged between 5 and 300 µm in SLM and 5 and 70 µm in EBM. The distribution of the porosity in the xy planes can be observed in Figure 6. The figures confirm the difference in porosity but also reveal differences in their distribution.

Generally, two types of pores exist in powder-based AM: spherical, gas-induced pores, and irregular-shaped, process-induced pores [7,30]. The first can occur due to the presence of entrapped gas in the powder particles during atomization, the latter mainly associated with non-optimal process parameters [30]. According to Figure 6 and results from the image analysis, pores in the EBM sample were mostly gas pore type, while in SLM both types were observed.

An interesting point to notice on the micrographs presented in Figure 6 were the pore patterns. In the EBM build sample, pores seemed to form randomly at both build direction and build planes, while in SLM, there was a clear tendency to form preferentially in both directions. In SLM most pores tended to form linearly in the build direction. In the build plane, pores occurred mostly at or near to the corners of a scanning “island” (Figure 6b,c). The reason for having such a pore formation pattern in SLM may be related to the scanning strategies in use. When the microstructure was observed after etching in the xy-plane of SLM build sample (Figure 6c), one can identify the used scanning strategy. The approximate size and locations of one “island” is shown in Figure 6c by a red square. As seen in Figure 6c, pores were indeed most prevalent at the “island” intersection or corner regions. One of the possible reasons for this is when the laser reaches the “island” border and starts to melt the next scan vector, a process associated pores, such as keyhole and lack of fusion might be formed. This problem can be mitigated or eliminated by (1) increasing the overlap between “islands”, (2) introducing a shift and tilt between layers, (3) adding a contour scan with lower energy density in each “island” just before or after its completion, (4) changing the scanning speed when the scan vector approaches the “island” border.

### 3.4. Microstructures of the Build Material

**Figure 7:**
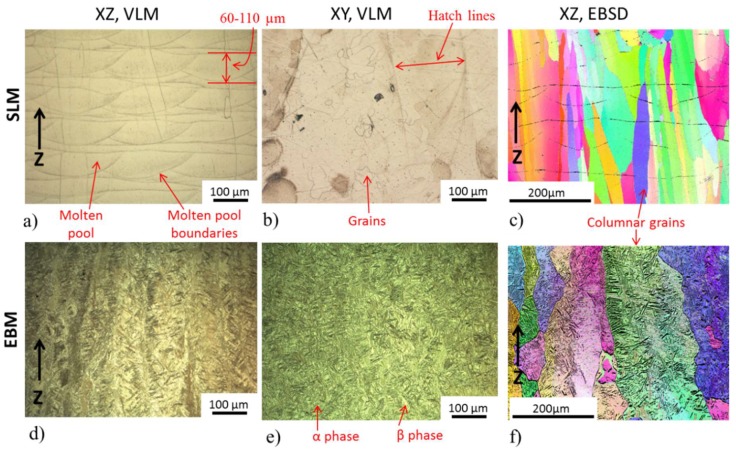
Microstructure of SLM (three images in first/top row) and EBM (all three images in second/bottom row) build samples. (**a**,**d**) are VLM image from the xz-plane; (**b**,**e**) are from the xy-plane; (**c**,**f**) are EBSD pattern from the xz-plane.

Figure 7 illustrates typical microstructural features from three perpendicular planes of SLM (first row) and EBM (second row) processed Ti55511 alloy. The orientation of the planes is indicated, and the axes are given in Figure 5b. In the xz surface plane of the SLM sample (Figure 7a), individual scan tracks and molten pool boundaries with the typical arc-shaped configuration are observed. The tracks are about 60–110 μm in thickness and 100–200 μm in width and are produced by the Gaussian-like energy distribution of the laser.

**Figure 8:**
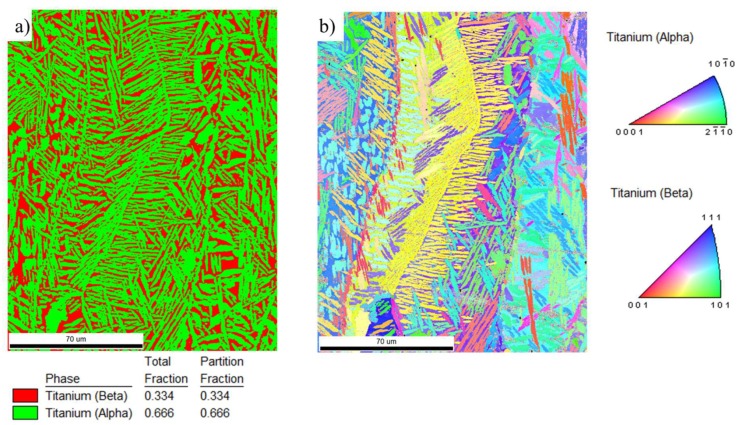
High-resolution EBSD (**a**) phase map and (**b**) IPF of EBM build sample.

Parallel to the building direction (Z) columnar grains are visible in both SLM and EBM samples. These columns are much larger than the layer thickness. The width of the columnar grains is smaller in the SLM built sample than in the EBM (Figure 7c,f). In the xy-plane the cross-section of the columns was equiaxed in both samples (Figure 7b,e). Despite significant alloy composition differences, in the EBM sample fine grains with lamella and Widmanstätten-like structure (Figure 7d–f) similar to EBM, processed Ti-6Al-4V can be observed within the large columns. A similar microstructure is observed in the used powder (Figure 3b). Figure 8 shows an EBSD phase map and an inverse pole figure taken from the xz-plane of the EBM sample revealing the presence of about 67 wt% α phase and 33 wt% β phase. Areas of similar orientation that were likely to have originated from the same parent β grain can be recognized.

This microstructure and phase differences between EBM and SLM build samples can be ascribed to the difference in build plate temperature and cooling rates applied. Because of faster scanning speed and a high chamber temperature of the EBM, the cooling rate will be slower than the SLM. Therefore, the average temperature of the melt pool and heat affected zone in EBM will be relatively higher than in SLM which will consequently lead to grain growth and β→α transformation.

Similar to the powder analysis, the chemical composition of the as-built samples was assessed by EDS analysis. The elemental map of the as-built SLM sample does not show any distinct regions and they all seem homogeneous and featureless. In the as-built EBM sample, however, there are two distinct regions in the elemental map like what was observed in the EBM powders after processing.

**Figure 9:**
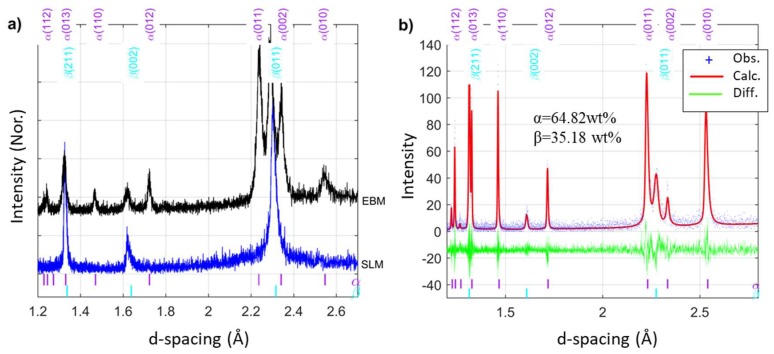
(**a**) Diffraction pattern of as-built samples. These color-coded tick marks under the diffractogram correspond to the expected peak positions of reported α- and β-Ti phases. (**b**) Rietveld refinement result of XRD data from EBM build sample.

The XRD analysis, performed on the xy-plane of the as-built samples, confirmed that the SLM consists predominantly of beta phase but reveals also the presence of a limited amount of α or α’ phase (<3 wt%) as shown in Figure 9a. Because of limited quantity and the almost identical unit cell parameters of α and α’ phase, it is not possible to distinguish between them. According to the literature, the α’ phase commonly associated with the extreme temperature change is usually observed in the SLM build Ti-6Al-4V [1,8,17]. The α phase, on the other hand, is associated with an isothermal cooling condition [2,10,17,31] and is commonly observed in the EBM build Ti-6Al-4V. Therefore, it is believed that that the minority phase present in the SLM build sample might be α’ phase.

The XRD pattern of the EBM sample confirms the presence of α and β phases. Rietveld analysis (Figure 9b) was performed and the lattice constants of hexagonal close packed α phase determined to be *a* = 2.931(7) Å and *c* = 4.658(8) Å, respectively, with *c/a* ratio of 1.5891. For the body-centered cubic β phase, *a* = 3.230(9) Å. As shown in the figure, the phase fraction of the α phase is 64.82 wt% and the β phase is 35.18%. This result supports the observation from the SEM.

The mechanical properties of a Ti-alloys are strongly determined by their microstructure and phase composition. Measured microhardness of various samples seemed to match with literature. The mean microhardness profiles of the SLM and EBM build samples were 315 ± 3.41 HV and 348 ± 30.20 HV, respectively. Increased microhardness of the EBM build sample can be attributed to the amount of α phase present. From the values of standard deviations of microhardness, it is evident that the SLM build sample has relatively consistent microhardness while the EBM samples show a larger deviation. This variation indicates that the SLM sample has a uniform microstructure throughout while the EBM sample has an inhomogeneous microstructure. This agrees well with the microstructure presented in Figure 7 and Figure 8. Here it is also important to point out that despite apparent differences in alloy chemical composition, the microhardness of the EBM build sample is very similar to α phase dominated EBM build Ti-6Al-4V reported by Neikter et al. [32]. Such similarity can be explained by the similar α + β microstructure observed in both types of alloys after the EBM process. The microhardness of the EBM build sample seemed to slightly lower (but same in standard deviation) than what Kurzynowski et al. [13] reported for EBM build Ti55511, previously. As hardness of the material is the resistance of the material to plastic deformation, one can expect that the EBM build sample may show higher strength than the SLM build samples. However, this needs further investigation.

## 4. Conclusions

The Ti55511 parts were successfully fabricated using SLM and EBM techniques. The results indicate that both methods can process Ti55511 and achieve almost full density with limited porosity. For the process parameters used in this study, the SLM process gives a slightly lower density and better surface quality. The shape of the pores in the EBM production appears to be mostly spherical while more random shapes are observed in SLM. The dominant β phase in the original powder becomes a minority phase after EBM processing, while there is almost no phase transformation in the SLM. Because of high build temperature and relatively slow cooling rate, the EBM build samples show a lamella and Widmanstätten-like structure similar to the microstructure observed in EBM processed Ti-6Al-4V despite it is alloy composition. Because of the lamella microstructure, the EBM build sample showed about 10% higher microhardness than the SLM build samples and it measured as 348 ± 30.20 HV. To achieve near-β phase composition in Ti55511 after EBM processing, the current processing route needs to be optimized or the build part needs additional post-heat treatments.

## Figures and Tables

**Table 1:** Powder size distribution before and after processing measured by the laser diffraction method.

	Ti55511					
	SLM			EBM		
	Before	After	% Change	Before	After	% Change
D_10_ (µm)	27.96	30.42	8.80	50.88	59.74	17.41
D_50_ (µm)	40.42	44.82	10.89	70.41	85.32	21.18
D_90_ (µm)	60.32	68.58	13.68	99.23	127	28.02

“D_10_”, “D_50_”, and “D_90_” mean the particle sizes at 10 vol%, 50 vol%, and 90 vol%, respectively.
